# Molecular mechanisms of calcium signaling in the modulation of small intestinal ion transports and bicarbonate secretion

**DOI:** 10.18632/oncotarget.23197

**Published:** 2017-12-11

**Authors:** Xin Yang, Guorong Wen, Biguang Tuo, Fenglian Zhang, Hanxing Wan, Jialin He, Shiming Yang, Hui Dong

**Affiliations:** ^1^ Department of Gastroenterology, Xinqiao Hospital, Third Military Medical University, Chongqing 400037, China; ^2^ Department of Gastroenterology, Affiliated Hospital, Zunyi Medical College, and Digestive Disease Institute of Guizhou Province, Zunyi 563003, China; ^3^ Department of Medicine, School of Medicine, University of California, San Diego, CA 92093, USA

**Keywords:** carbachol, Ca^2+^ signaling, SOCE, duodenal epithelial ion transports

## Abstract

**Background and Purpose:**

Although Ca^2+^ signaling may stimulate small intestinal ion secretion, little is known about its critical role and the molecular mechanisms of Ca^2+^-mediated biological action.

**Key Results:**

Activation of muscarinic receptors by carbachol(CCh) stimulated mouse duodenal *I*_sc_, which was significantly inhibited in Ca^2+^-free serosal solution and by several selective store-operated Ca^2+^ channels(SOC) blockers added to the serosal side of duodenal tissues. Furthermore, we found that CRAC/Orai channels may represent the molecular candidate of SOC in intestinal epithelium. CCh increased intracellular Ca^2+^ but not cAMP, and Ca^2+^ signaling mediated duodenal Cl^-^ and HCO_3_^-^ secretion in wild type mice but not in CFTR knockout mice. CCh induced duodenal ion secretion and stimulated PI3K/Akt activity in duodenal epithelium, all of which were inhibited by selective PI3K inhibitors with different structures. CCh-induced Ca^2+^ signaling also stimulated the phosphorylation of CFTR proteins and their trafficking to the plasma membrane of duodenal epithelial cells, which were inhibited again by selective PI3K inhibitors.

**Materials and Methods:**

Functional, biochemical and morphological experiments were performed to examine ion secretion, PI3K/Akt and CFTR activity of mouse duodenal epithelium. Ca^2+^ imaging was performed on HT-29 cells.

**Conclusions and Implications:**

Ca^2+^ signaling plays a critical role in intestinal ion secretion via CRAC/Orai-mediated SOCE mechanism on the serosal side of epithelium. We also demonstrated the molecular mechanisms of Ca^2+^ signaling in CFTR-mediated secretion via novel PI3K/Akt pathway. Our findings suggest new perspectives for drug targets to protect the upper GI tract and control liquid homeostasis in the small intestine.

## INTRODUCTION

Epithelial ion transports are critical physiological processes in the human gastrointestinal (GI) tract. Intestinal epithelium either absorbs electrolytes or secretes ions (such as Cl^-^ and HCO_3_^-^), which provides the driving force for water absorption or secretion to maintain the liquid homeostasis in the human body. Epithelial ion transports are under control of several neuro-humoral factors, including ACh, 5-HT, PGs, nitric oxide (NO), and capsaicin-sensitive afferent neurons [1]. These neuro-humoral factors mediate epithelial ion transports through three major cellular signaling: Ca^2+^-, cAMP- and cGMP-dependent pathways. Among them, ACh is one of the major excitatory neurotransmitter in GI system. The chemical analogue of ACh, carbachol (CCh), a muscarinic receptor agonist, is a commonly used Ca^2+^ mobilizer. Currently, the physiological roles and molecular mechanisms of cAMP- and cGMP-dependent regulation of epithelial Cl^-^ and HCO_3_^-^ secretion are relatively well defined; however, those mediated by Ca^2+^ signaling remain poorly understood in small intestinal epithelia [2].

Moreover, although it is known that Ca^2+^ signaling is critical for intestinal epithelial ion secretion [2], the underlying detailed mechanisms that control cytosolic Ca^2+^ concentration ([Ca^2+^]_cyt_) homeostasis in small intestinal epithelium are not fully understood [3]. It is generally thought that agonists induce Ca^2+^ signaling via two major processes in non-excitable cells: the IP_3_-induced release of Ca^2+^ from intracellular stores, and then an enhanced Ca^2+^ entry from the extracellular medium [4]. Classically, the Ca^2+^ entry in non-excitable epithelial cells was thought to occur mainly via so-called capacitative or store-operated Ca^2+^ channels (SOCs), which activation is entirely dependent on the depletion of intracellular Ca^2+^ stores [5, 6]. These channels are the Ca^2+^-release activated Ca^2+^ channels (CRAC) first described in mast cells and Jurkat lymphocytes [7, 8], which are accomplished by the pore forming Ca^2+^ channel Orai [9, 10]. However, only a few studies have focused on the role of CRAC/Orai channels in polarized epithelial cells, such as intestinal epithelial cell line IEC-6 cells [11] and colonic epithelial cells [12, 13]. So far, the regulatory mechanisms of [Ca^2+^]_cyt_ homeostasis in native epithelial cells of the small intestine are still unclear.

The major focus of Cl^-^ and of HCO_3_^-^ secretion in the small intestine is on cAMP- and cGMP-dependent regulatory pathways, which mediates several membrane ion channels to contribute to epithelial ion transports. The cystic fibrosis transmembrane conductance regulator (CFTR) is one of the critical channels in the luminal membrane of enterocytes [14]. CFTR is a cAMP/PKA and cGMP/PKG -dependent channel abundantly expressed in several functionally diverse tissues, such as the pancreas, intestine, kidney, sweat duct, and lung [15, 16]. In intestinal epithelial cells (IEC), it regulates Na^+^, Cl^-^, and HCO_3_^-^ transports [17]. Although CFTR is principally activated by cAMP/PKA and cGMP/PKG pathways, it is also regulated by Ca^2+^ signaling [18]. However, it is currently unclear for the detailed regulatory mechanisms of CFTR by Ca^2+^ signaling, particularly it is unknown if Ca^2+^ signaling per se is able to activate CFTR or is through potentiating cAMP/PKA-mediated CFTR activation.

The duodenal mucosa, due to its strategic location between the stomach and other segments of the small intestine, senses luminal nutrients and regulates duodenal epithelial ion transports, particularly Cl^-^ and HCO_3_^-^ secretion, which in turn is important for nutrient absorption and mucosal protection from gastric acid [19]. Therefore, in the present study, we sought to investigate Ca^2+^ signaling mediated duodenal epithelial ion secretion and the underlying molecular mechanisms. We found that CCh stimulates extracellular Ca^2+^ entry likely through SOC/Orai channels mainly from the basolateral membrane of IEC. The increased [Ca^2+^]_cyt_ per se is enough to trigger duodenal transepithelial secretion through a novel PI3K/AKT/CFTR pathway. This study not only reveals that Ca^2+^ signaling is critical to activate CFTR-mediated epithelial ion transportsin the small intestine, but also provides novel insights into the detailed mechanisms of Ca^2+^-dependent transepithelial Cl^-^ and HCO_3_^-^ secretion.

## RESULTS

### Activation of muscarinic receptors induced intracellular Ca^2+^ release and extracellular Ca^2+^ entry

We applied CCh, a muscarinic receptor agonist, to mobilize intracellular Ca^2+^. CCh (100 μM) markedly increased duodenal short-circuit current (*I*_sc_)(Figure 2A), which was attenuated by atropine(10 μM), a muscarinic receptor antagonist (Supplementary Figure 1A). Activation of muscarinic receptors stimulates the production of inositol 1,4,5-trisphoshate (IP_3_), leading to intracellular Ca^2+^ release via IP3 receptors on the endoplasmic reticulum (ER) membrane [20]. We used LiCl(30mM) to inhibit IP_3_ production and confirmed the involvement of IP_3_ pathway in the process of muscarinic receptors-mediated intracellular Ca^2+^ release (Supplementary Figure 1B).

**Figure 1:**
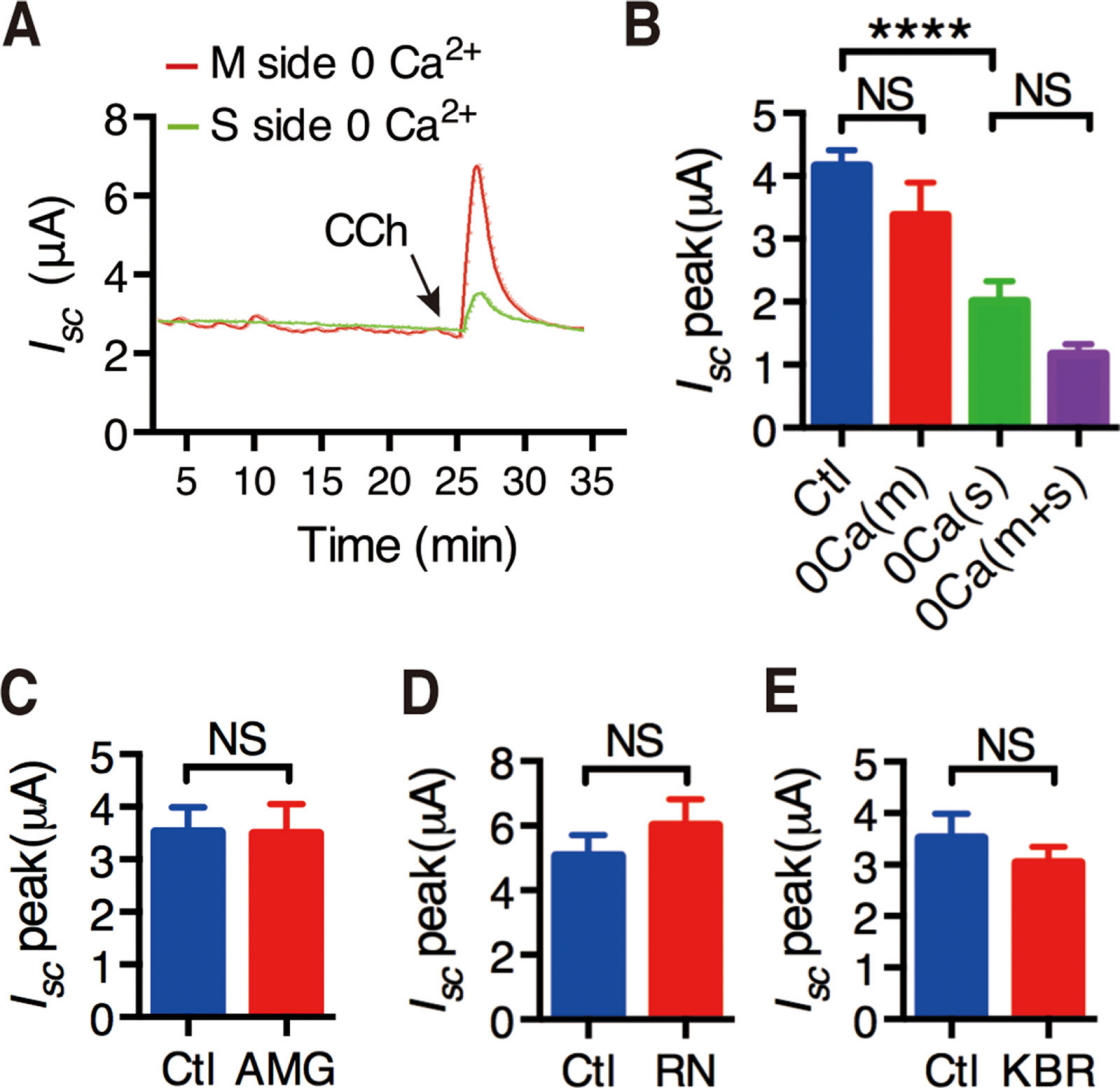
Activation of muscarinic receptors induced extracellular Ca^2+^ entry through possible pathways (**A**) Representative of the time course of CCh-stimulated murine duodenal mucosal *I*_sc_ after extracellular Ca^2+^ was omitted from the serosal side or the mucosal side of the duodenal tissues. (**B**) The summary data of CCh-stimulated duodenal *I*_sc_ peak after Ca^2+^ omission from the mucosal side, the serosal side or both sides of duodenal tissues. (**C**–**E**) Effects of AMG-517 (AMG, 100 M) (C), RN-1734 (RN, 3 M) (D) and KBR-7943(KBR, 3 M) (E) on CCh-induced duodenal *I*_sc_ peak after serosal addition. Results are presented as mean SE (*n =* 6–9 in each series). NS, no significant differences, ^****^P < 0.0001 *vs.* control by Student’s *t*-test.

To further test if intracellular Ca^2+^ release mediates extracellular Ca^2+^ entry into IEC, CCh-stimulated duodenal *I*_sc_ was compared in the presence or the absence of extracellular Ca^2+^ in each side of the Ussing chamber experiments. As shown in Figure 1A and 1B, CCh-stimulated duodenal *I*_sc_ was significantly attenuated when extracellular Ca^2+^ was omitted from the serosal side of the duodenal tissues, but not from the mucosal side. Moreover, CCh-stimulated duodenal *I*_sc_ was not significantly different between Ca^2+^ omissions from the serosal side only and from both sides of the tissues (Figure 1B). Therefore, CCh may induce intracellular Ca^2+^ release that further mediates extracellular Ca^2+^ entry from the serosal side of the duodenal epithelium.

### CCh induced SOCE mechanism on the serosal side of the duodenal epithelium

**Figure 2:**
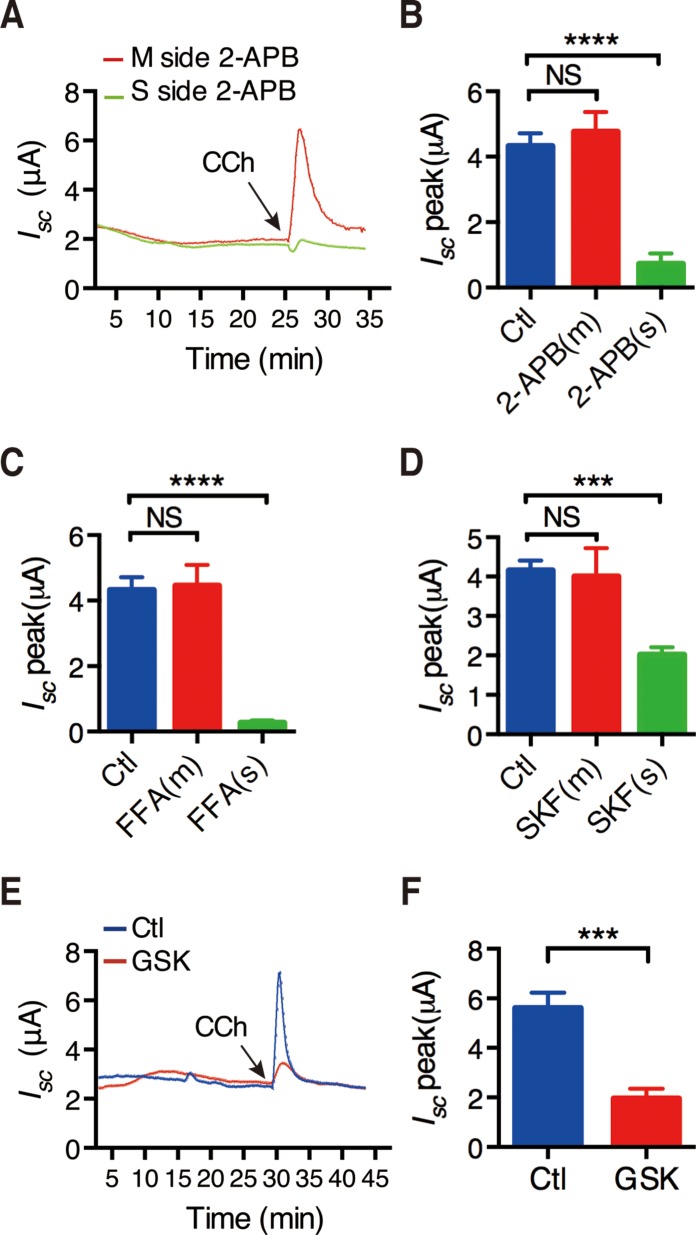
CRAC/Orai channels in the regulation of CCh-stimulated duodenal *I*_sc_ (**A**) Representative of the time course of CCh -stimulated murine duodenal *I*_sc_ with or without addition of 2-aminoethoxydiphenyl borate (2-APB, 50 M) to the mucosal or serosal side of duodenal tissues. (**B**) The summary of the effect of 2-APB on CCh-stimulated murine duodenal *I*_sc_ peak after mucosal or serosal addition. (**C**–**D**) Summery effects of the flufenamic acid (FFA, 100 M) (C) or SKF-96365 (SKF, 3 M) (D) on CCh-stimulated duodenal *I*_sc_ peak after mucosal or serosal addition. (**E**) Representative of the time course of CCh-stimulated duodenal *I*_sc_ with or without serosal addition of GSK-7975A (GSK, 100 M). (**F**) The summary of the effect of GSK-7975A on CCh-stimulated duodenal *I*_sc_ peak after serosal addition. Results are presented as mean SE (*n =* 6–9 in each series). NS, no significant differences, ^***^P < 0.001, ^****^P < 0.0001 *vs.* control.

It is well known that intracellular Ca^2+^ release to deplete the Ca^2+^ store in the ER would promote extracellular Ca^2+^ entry, which is the so-called store-operated Ca^2+^ entry (SOCE). Because it is still uncertain for the functional expression of the voltage-operated Ca^2+^ channels in small intestinal epithelia, it is believed that SOCE fulfill this function [21]. To test if this mechanism occurs in duodenal epithelium, we used three selective SOCE blockers with different chemical structures. As shown in Figure 2A, addition of 2-Aminoethoxydiphenyl borate (2-APB) (50 μM) to the mucosal side of the duodenal tissues did not affect the time course of CCh-stimulated duodenal *I*_sc_, but addition to serosal side significantly suppressed the duodenal *I*_sc_. Figure 2B summarizes the effect of 2-APB on duodenal *I*_sc_ peak after it was added to each side of the duodenal tissues. Similar to 2-APB, both Flufenamic acid (FFA)(100 μM) and SKF-96365 (30 μM) significantly suppressed duodenal *I*_sc_ peak from serosal side of the duodenal tissues, but not from the mucosal side (Figure 2C and 2D). Therefore, SOCE mechanism occurs exclusively on the serosal side of the duodenal epithelium, which is consistent with the previous findings described above.

### CRAC/Orai channels in the regulation of CCh-stimulated duodenal *I*_sc_

We screened the molecular candidates of SOCE in duodenal epithelium. We first examined common Ca^2+^-permeable channels, such as TRPV1, TRPV4 and NCX using their selective blockers AMG-517 (100 μM), RN-1734 (30 μM) and KBR-7943 (30 μM), respectively. As shown in Figure 1C, 1D and 1E, addition of them to the serosal side did not alter CCh-induced duodenal *I*_*sc*_, indicating no functional expression of TRPV1, TRPV4 and NCX to exclude them as the molecular candidates of SOCE in duodenal epithelium.

We further identified the molecular candidates of SOCE using GSK-7975A, a specific CRAC/Orai channel blocker. As shown in Figure 2E, addition of GSK-7975A(100 μM) to serosal side of the duodenal tissues significantly suppressed the time course of CCh-stimulated duodenal *I*_sc_. Figure 2F summarizes the inhibitory effect of GSK-7975A on duodenal *I*_sc_ peak after it was added to serosal side. Therefore, CRAC/Orai channels may represent the molecular candidate of SOCE in duodenal epithelium.

### CRAC/Orai channels in CCh-mediated Ca^2+^ entry into IEC

**Figure 3:**
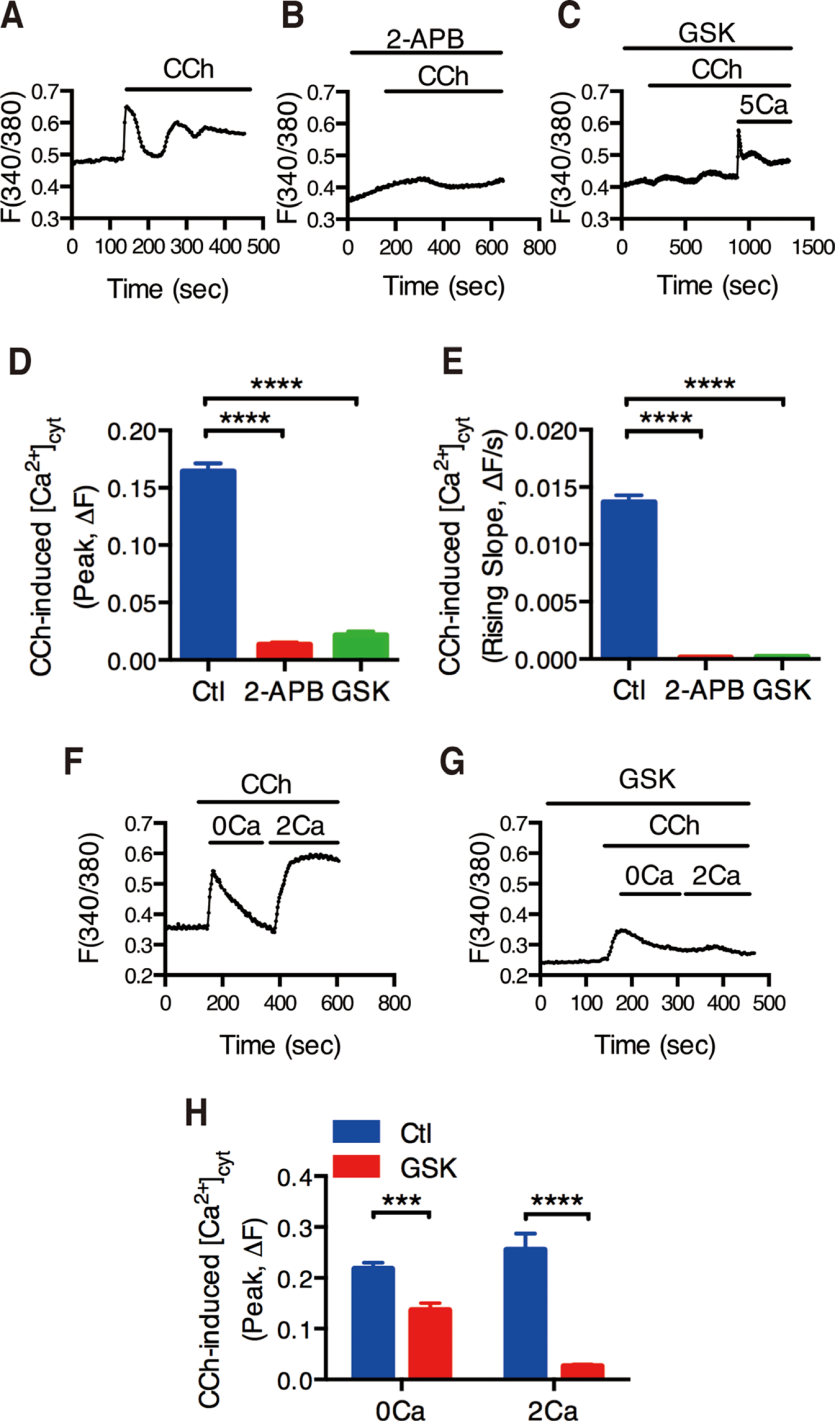
Functional identification of CRAC/Orai channels in CCh-mediated Ca^2+^ mobilization of intestinal epithelial cells (**A**) Time courses showing the effect of CCh (10 M) on basal [Ca^2+^]_cyt_ of human colon carcinoma HT-29 cells in normal physiological salt solutions. (**B**–**C**) Time courses showing the effect of 2-APB (5 M, B) or GSK-7975A (GSK, 3 M, C) on CCh-mediated [Ca^2+^]_cyt_ mobilization in HT-29 cells. (**D**–**E**) The summary of the effect of 2-APB or GSK on the peaks (D) and the rising slopes (F) of CCh-induced [Ca^2+^]_cyt_ signaling in HT-29 cells. (**F**) Time courses showing CCh (10 M) first caused a rapid increase in [Ca^2+^]_cyt_ when HT-29 cells was superfused with Ca^2+^-free solution (0 Ca) (*left*). After Ca^2+^ release from the ER was complete, restoration of extracellular Ca^2+^ (2 mM Ca) caused an additional increase in [Ca^2+^]_cyt_ in HT-29 cells (*right*). (**G**) Time courses showing the effect of GSK-7975A (GSK, 3 M) on CCh-induced [Ca^2+^]_cyt_ mobilization in HT-29 cells superfused with Ca^2+^-free solution (0 Ca) or Ca^2+^-containing solution (2 Ca). (**H**) Summary data on the effect of GSK-7975A on CCh-induced [Ca^2+^]_cyt_ mobilization in HT-29 cells superfused with Ca^2+^-free solution (0 Ca) or Ca^2+^-containing solution (2 Ca). Results are presented as mean SE (*n =* 20-30 cells). ^***^P < 0.001, ^****^P < 0.0001*vs.* control or DMSO.

Since HT-29 cell line is commonly used as a cell model of IEC to study ion absorption and secretion [22–24], and they express the muscarinic receptor [12, 25, 26], we used them to measure [Ca^2+^]_cyt_ by digital Ca^2+^ imaging. CCh(100 μM) immediately induced Ca^2+^ signaling in HT-29 cells (Figure 3A). 2-APB (50 μM) and GSK-7975(30 μM) almost abolished CCh-induced Ca^2+^ signaling (Figure 3B and 3C). Both peak and the rising slope of CCh-induced [Ca^2+^]_cyt_ signaling were significantly decreased by 2-APB and GSK-7975 (Figure 3D and 3E).

We further examined the role of CRAC/Orai channels in SOCE mechanism. In HT-29 cells superfused with Ca^2+^-free solution (0Ca) first caused a rapid increase in [Ca^2+^]_cyt_ due to intracellular Ca^2+^ release from the ER to the cytosol (Figure 3F, *left*). After Ca^2+^ release from the ER was complete, restoration of extracellular Ca^2+^ (2Ca) caused an additional increase in [Ca^2+^]_cyt_ due to SOCE mechanism (Figure 3F, *right*). As shown in Figure 3G and H, GSK-7975A (30 μM) significantly inhibited CCh-induced SOCE mechanism. Therefore, these data further support our previous notion that CRAC/Orai channels are the molecular candidate of SOCE in IEC.

### Role of CFTR channels in CCh-induced epithelial ion secretion

**Figure 4:**
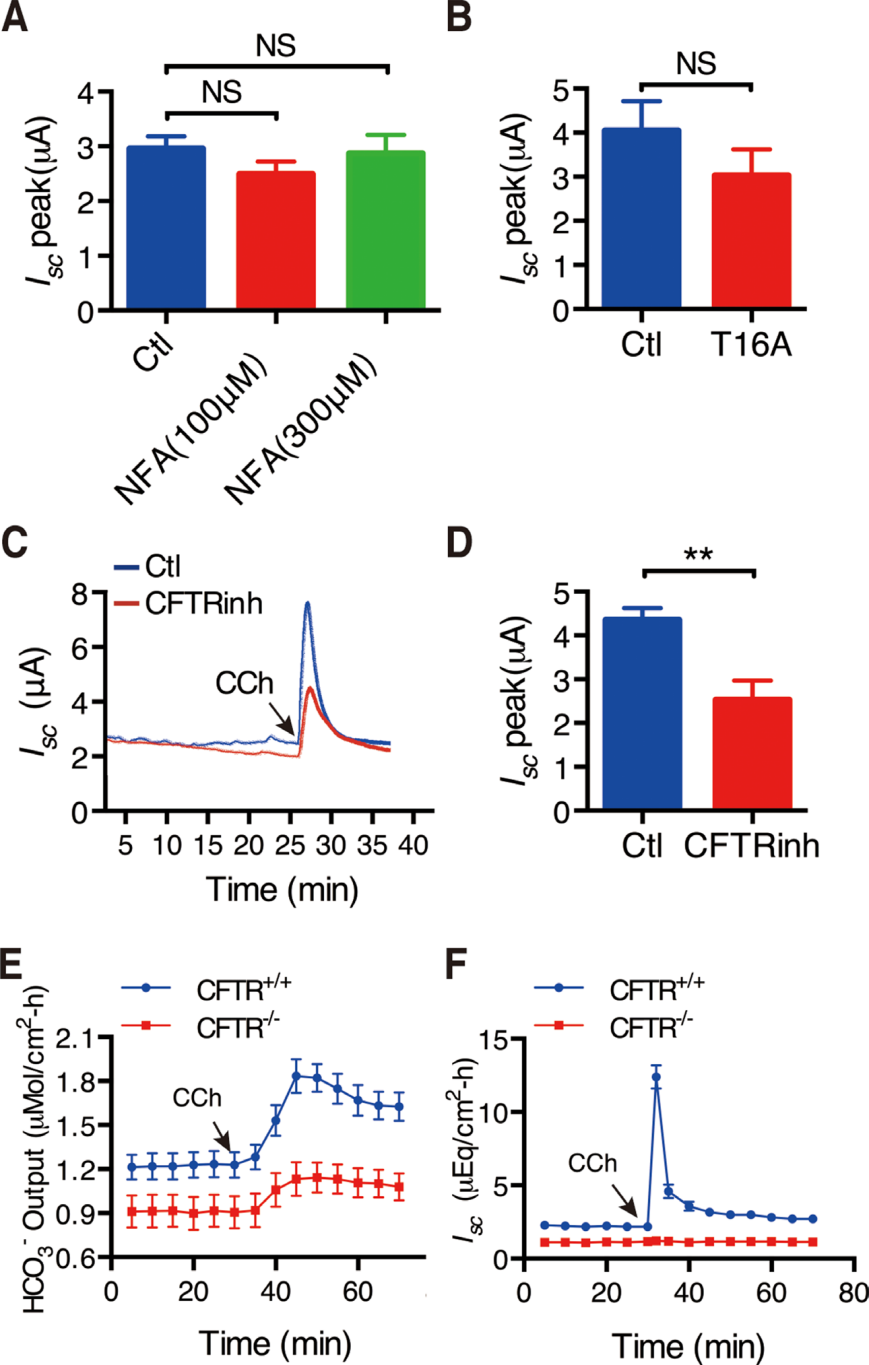
Role of CFTR channels in CCh-induced duodenal epithelial ion secretion (**A**–**B**) Summary data on the effects of NFA (10 M, *n =* 15 and 3 M, *n =* 6) (A) and T16Ainh-A01 (T16A, 30 M, *n =* 11) (B) on CCh-stimulated duodenal *I*_sc_ peaks after mucosal addition. (**C**) Representative of the time course of CCh-stimulated duodenal *I*_sc_ with or without mucosal addition of CFTR_inh_-172 (CFTR_inh_, 3 M). (**D**) Summary on the effect of CFTR_inh_-172 (CFTR_inh_, 3 M) on CCh-stimulated duodenal *I*_sc_ peak (*n =* 9). (**E**–**F**) Summary on the time courses of CCh-stimulated duodenal HCO_3-_ secretion (E) and *I*_sc_ (F) in CFTR+/+ or CFTR^-/-^ mice (*n =* 6). Results are presented as mean SE. NS, no significant differences, ^**^P < 0.01*vs.* control.

First, to test if CCh-induced Ca^2+^ signaling could stimulate duodenal *I*_sc_ through Ca^2+^-activated Cl^-^ channels (CaCC), we applied commonly used CaCC blocker niflumic acid (NFA). At the concentrations of 100-300 μM, it did not affect CCh-induced duodenal *I*_sc_ (Figure 4A). Furthermore, T16A_inh_-A01(300 μM), a selective potent CaCC blocker did not affect CCh-induced duodenal *I*_*sc*_ either (Figure 4B). These results exclude the role of CaCC in the CCh-induced duodenal epithelial ion secretion.

Second, we examined the role of CFTR channels in this process since they are critical in epithelial ion secretion stimulated by several secretagogues. As shown in Figure 4C and 4D, CFTR_inh_-172 (30 μM), a highly potent and specific CFTR inhibitor [27], markedly inhibited CCh-induced duodenal *I*_sc_. Further, we applied CCh to CFTR^+/+^ and CFTR^-/-^ mice, and found that CCh markedly stimulated duodenal *I*_*sc*_ and HCO_3_^-^ secretion in CFTR^+/+^ mice (Figure 4E and 4F). However, CCh failed to induce duodenal *I*_*sc*_ and decreased duodenal HCO_3_^-^ secretion in CFTR^-/-^ mice. The net peak of CCh-stimulated duodenal HCO_3_^-^ secretion was reduced by 61% and the net peak of duodenal *I*_*sc*_ was reduced by 99%, respectively in CFTR^-/-^ mice (Figure 4E and 4F). We therefore underscored the critical role of CFTR channels in CCh-induced Ca^2+^-mediated duodenal ion secretion.

### CCh-induced duodenal ion secretion was cAMP/PKA-independent

Since Ca^2+^ signaling is able to activate CFTR-mediated ion secretion through cAMP/PKA pathway in other epithelial tissues, we tested this notion in CCh-induced duodenal epithelial ion secretion. We first determined if there is a cross-talk between Ca^2+^ and cAMP signaling in the activation of CFTR channels. When low concentrations of cAMP-generating agonist forskolin(0.15 μM) and CCh (30 μM) were added together, a synergistic effect on duodenal *I*_*sc*_ was observed (the green line and bar in Supplementary Figure 2A and 2B). However, this synergistic effect was not affected by the pretreatment of H89(20 μM), a commonly used PKA inhibitor (Supplementary Figure 2D–2F).

To exclude the role of cAMP/PKA pathway in CCh-induced duodenal ion secretion, we directly measured cAMP activity. As shown in Supplementary Figure 2C, CCh (100 μM) did not alter cAMP concentration in mouse duodenal epithelium, but forskolin (10 μM) markedly increased it. These results further confirm that [Ca^2+^]_cyt_-mediated duodenal ion secretion is cAMP/PKA-independent although a synergy exists between these two signaling pathways.

### PI3K/Akt in CCh-induced duodenal ion secretion

**Figure 5:**
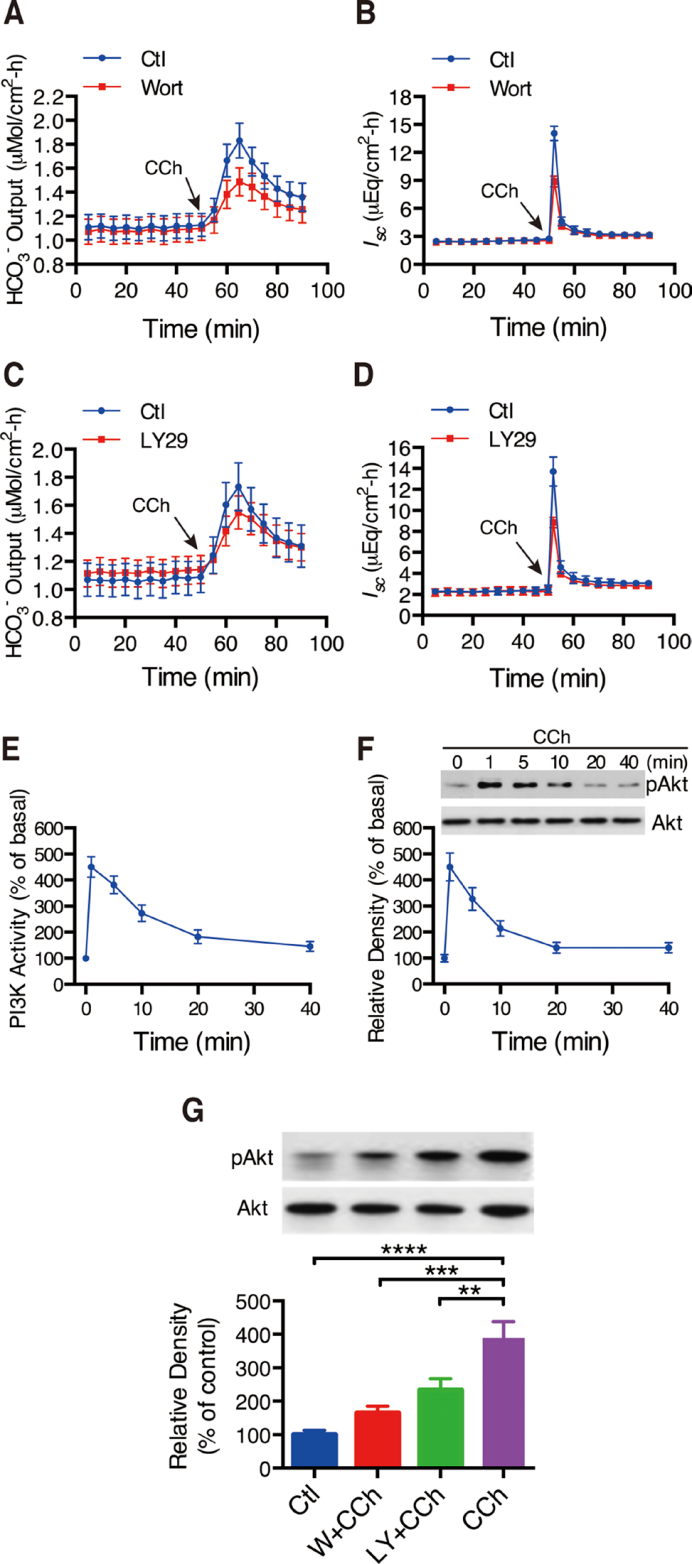
Involvements of PI3K/Akt in CCh-induced duodenal ion secretion (**A**–**B**) Summary on the time courses of CCh-stimulated duodenal HCO_3-_ secretion (A) and *I*_sc_ (B) with or without wortmannin (Wort, 0.1 M) added to serosal side (*n =* 7). (**C**–**D**) Summary on the time courses of CCh-stimulated duodenal HCO_3-_ secretion (A) and *I*_sc_ (B) with or without LY294002 (LY29, 2 M) added to serosal side (*n =* 7). (**E**) Time course of CCh-stimulated duodenal mucosal epithelial PI3K activity. Murine duodenal mucosa was treated for various periods of time with CCh(10 M). Mucosal extract was immunoprecipitated with anti-PI3K P85 antibody *in vitro* (*n =* 4). (**F**) Time course of CCh-stimulated phosphorylation of Akt. Duodenal mucosae were incubated with CCh for the indicated times and were subjected to Western blot analysis. *Top*: blots showing Akt phosphorylation. Data are from a single experiment representative of 4 experiments. *Bottom*: Summary results are expressed as the percentage of basal values (*n =* 4). (**G**) Effects of wortmannin (W, 0.1 M), or LY294002 (LY, 2 M) on CCh-stimulated phosphorylation of Akt of murine duodenal mucosa. The summary results are expressed as the percentage of controls (*n =* 4). Results are presented as mean SE. ^**^p < 0.01, ^***^P < 0.001, ^****^P < 0.0001 *vs.* control.

Growing evidence suggest that CFTR channels can be activated by Ca^2+^-dependent PKA, PKC and tyrosine kinase in different epithelial tissues [28]. Here we examined if PI3K/Akt is involved in CCh-induced duodenal ion secretion. As shown in Figure 5A–5D, both selective PI3K inhibitors, wortmannin (0.1 μM) and LY294002 (20 μM), which have been shown to target PI3K activity at these concentrations [29, 30] significantly reduced CCh-stimulated mouse duodenal HCO_3_^-^ secretion and duodenal *I*_*sc*_. Wortmannin reduced net peak of CCh-stimulated duodenal HCO_3_^-^ secretion by 49% and duodenal *I*_*sc*_ by 42%, respectively (Figure 5A and 5B). LY294002 reduced net peak of CCh-stimulated duodenal HCO_3_^-^ secretion by 23% and duodenal *I*_*sc*_ by 43%, respectively (Figure 5C and 5D).

To confirm the role of PI3K in the regulation of CFTR function, PI3K activity in duodenal epithelium was measured. CCh (100 μM) rapidly stimulated PI3K activity and reached the peak within 1 min (Figure 5E). CCh induced the maximal PI3K activity by 4.5-fold compared with basal levels. Subsequently, we further examined whether CCh induces phosphorylation of Akt, a downstream effector of PI3K. Likewise, CCh caused a rapid phosphorylation of Akt. Notably, the time courses of CCh-stimulated PI3K activity and phosphorylation of Akt in duodenal epitheliumare matchable (Figure 5F). Again, both wortmannin and LY294002 significantly inhibited CCh-stimulated phosphorylation of Akt (Figure 5G). This direct evidence confirms the critical role of PI3K/Akt in CCh-induced Ca^2+^-mediated activity of CFTR channels.

### PI3K/Akt in CCh-mediated phosphorylation and translocation of CFTR channels

The phosphorylation and translocation of CFTR channels to the surface of duodenal villus cells are important indicators of CFTR activity. To provide the confirmatory evidence for the role of PI3K in Ca^2+^ signaling mediated CFTR activation, we assessed CCh-stimulated translocation and phosphorylation of CFTR channels in duodenal epithelial cells.

**Figure 6:**
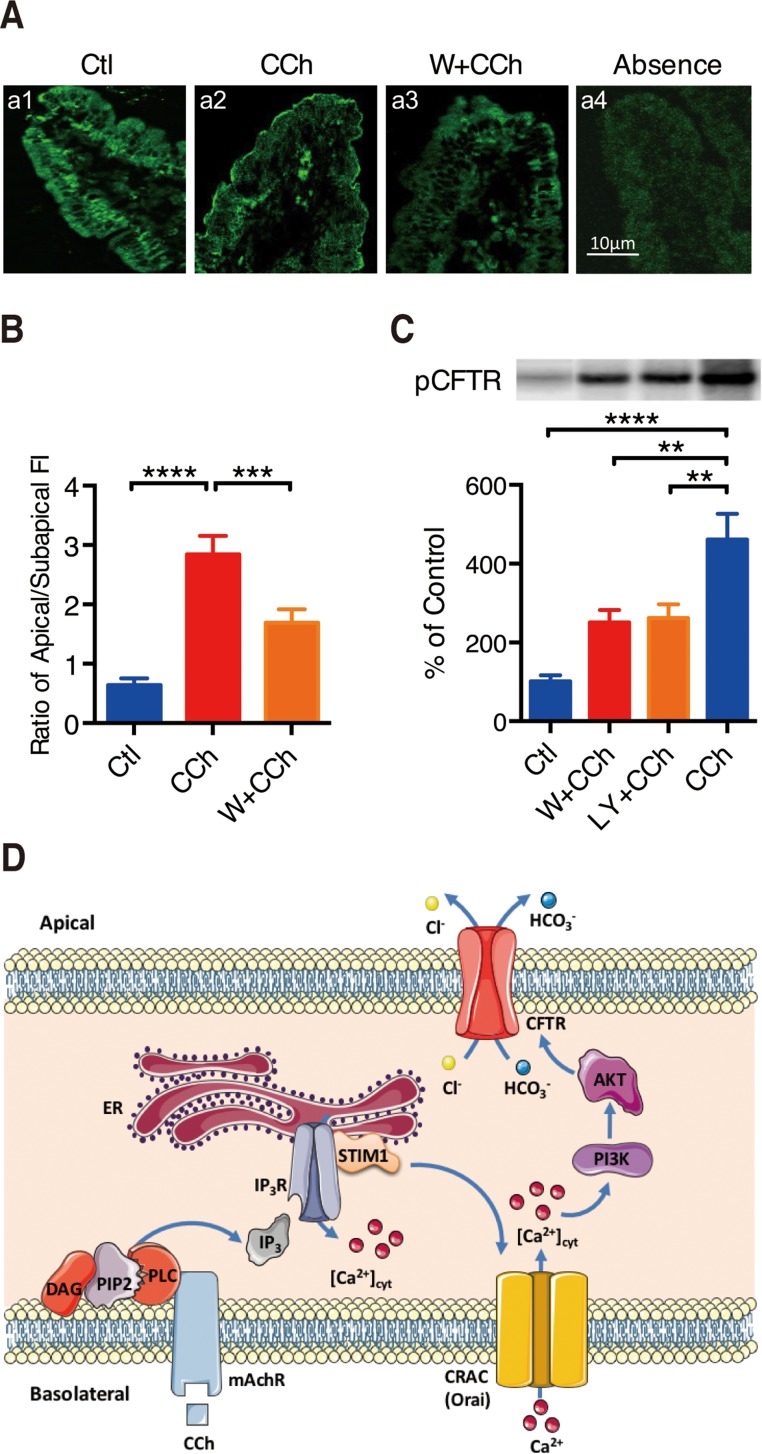
PI3K/Akt in CCh-mediated phosphorylation and translocation of CFTR channels (**A**) Representative showing effect of wortmannin (W, 0.1 M) on CCh-induced CFTR trafficking to the plasma membranes of duodenal villus cells. *a1*: distribution of CFTR in unstimulated control vehicle-treated duodenal villus cells. *a2*: distribution of CFTR in duodenal villus cells at 5 min after stimulation using CCh (100 M). *a3*: effect of wortmannin (W, 0.1 M) on CCh-induced distribution of CFTR in duodenal villus cells. *a4*: control with CCh in the absence of primary anti-CFTR antibody. Magnification, 600; calibration bar, 10 m. (**B**) Summary data on the ratio of plasma membrane to cytoplasm CFTR fluorescence intensity (FI). Values are mean SE (*n =* 120 cells in 4 animals, 30 cells from each tissue). (**C**) Summary data on the effects of wortmannin (W, 0.1 M), LY294002 (LY, 2 M) on CCh-induced CFTR phosphorylation in duodenal mucosal epithelial cells. *Top*: autoradiographs showing 32P-phosphorylated CFTR. Data are from a single representative experiment of 4 experiments in each series. *Bottom*: The summary results are expressed as a percentage of controls. Values are mean SE (*n =* 4). (**D**) Schematic diagram depicting the proposed mechanisms of Ca^2+^-mediated duodenal epithelial HCO_3-_ and Cl^-^ secretion mediated by CCh.

We first examined the effect of CCh on translocation of CFTR to the surface of duodenal villus cells in mice. As shown in Figure 6A, CFTR proteins were mainly located in the cytoplasmic compartments of duodenal villus cells in control (a1 in Figure 6A). However, after administration of CCh (100 μM) for 5 min, CFTR proteins were prominent along the plasma membranes of villus cells (a2 in Figure 6A), indicating Ca^2+^ signaling-stimulated trafficking of CFTR proteins to the plasma membranes of villus cells. Wortmannin(0.1 μM) markedly inhibited CCh-induced CFTR redistribution from the cytoplasmic compartments to the plasma membranes (a3 in Figure 6A). After quantifying the ratio of CFTR fluorescence intensity in plasma membrane and cytoplasm, CCh increased the ratio by 4.4-fold compared with control, but wortmannin decreased CCh-increased ratio by 41% (Figure 6B). Therefore, CCh-induced Ca^2+^ signaling stimulates translocationof CFTR to the surface of duodenal villus cells, in which PI3K plays a critical role.

We further examined the role of CCh-induced Ca^2+^ signaling in CFTR phosphorylation of mouse duodenal epithelia. The upper panel in Figure 6C illustrates a representative autoradiograph of CFTR phosphorylation after pretreatment with CCh and CCh plus either wortmannin (0.1 μM) or LY294002(20 μM). The lower panel in Figure 6C summarizes the intensity of CFTR phosphorylation. Compared with control, CCh increased CFTR phosphorylation by 4.6-fold. However, wortmannin and LY294002 inhibited CCh-induced CFTR phosphorylation by 46% and 43%, respectively. Therefore, these data not only confirm CCh-induced Ca^2+^ signaling stimulates CFTR phosphorylation, but also indicate the important role of PI3K in the activation of duodenal epithelial CFTR channels.

## DISCUSSION AND CONCLUSIONS

Although it is known for the importance of Ca^2+^ signaling in ion transports in the salivary gland, pancreatic ducts and colonic epithelia, the detailed role of [Ca^2+^]_cyt_ in the modulation of samll intestinal epithelial ion secretion and the underlying molecular mechanisms are not fully understood. In the present study, using native mouse duodenal epithelium with preserved polarity we reveal that: 1) activation of muscarinic receptors induces epithelial ion secretion via a pure Ca^2+^ signaling rather than a combination to cAMP/PKA pathway; 2) muscarinic receptor-induced Ca^2+^ signaling is via the SOCE mechanism on the serosal side of epithelial cells; 3) CRAC/Orai channels may serve as SOCE mechanism to mediate Ca^2+^-dependent ion secretion; 4) Ca^2+^ signaling regulates ion secretion via a novel PI3K/Akt-mediated activity of CFTR channels. Therefore, our results not only indicate that Ca^2+^ signaling per se plays a critical role in the regulation of small intestinal ion transports, but also provide a novel insight into the molecular mechanisms of Ca^2+^-mediated epithelial ion secretion.

[Ca^2+^]_cyt_ acts as an universal second messenger to regulate many different cellular functions in a variety of cells [31], including epithelial cells, and [Ca^2+^]_cyt_ is considered as an important regulator of intestinal ion transports [2, 32, 33]. In excitable cells, Ca^2+^ entry is mainly mediated via voltage-gated Ca^2+^ channels (VGCC). However, little is known about Ca^2+^ entry pathways in nonexcitable intestinal epithelial cells, since they may not express functional VGCC [34]. There was essentially less information available about the specific Ca^2+^ entry pathways existing in native small intestinal epithelium to mediate ion secretion. Therefore, in the present study, we firstly confirmed the critical role of pure Ca^2+^ signaling in duodenal ion secretion. Since intestinal epithelium is polarized in nature, we then revealed that CCh-induced external Ca^2+^ entry is just from serosal side of duodenal epithelial cells rather than from the lumen. We also examined the potential molecular candidate of SOCE mechanisms in regulating duodenal ion transport, and identified CRAC/Orai channels as the most likely molecular candidate, which is partially consistent with a previous report that STIM1/Orai may regulate Ca^2+^ influx across the apical and basolateral membrane in rat colonic epithelium [12]. However, it is currently unknown why CCh induces Ca^2+^ influx across the basolateral membrane only in mouse duodenal epithelium, which needs further investigation.

cAMP and [Ca^2+^]_cyt_ signaling are pleiotropic primary second messengers that regulate all secretory epithelia functions. Since cAMP pathway has been extensively studied, it is questioned if pure Ca^2+^ signaling is critical for intestinal ion secretion. Moreover, mutual regulation of cAMP and Ca^2+^ signaling is referred to as crosstalk, while integration of their effects can result in an additive or synergistic physiological response [35]. So far, the synergism of cAMP and Ca^2+^ signaling has been demonstrated for ion secretion in salivary gland and pancreatic ducts [36], but not in small intestinal epithelium. The available findings suggest that the IP3R binding protein released with inositol 1,4,5-trisphosphate (IRBIT) protein is a central component of the mechanism mediating the synergism between cAMP and Ca^2+^ by functioning as a third messenger that translocates between the IP_3_R and target proteins [37, 38]. Both cAMP and PKA are critical stimulators for this synergistic process. However, in the present study, although we observed the synergism of cAMP- and Ca^2+^-mediated ion secretion, we did not detect any change in cAMP concentrations induced by CCh in duodenal epithelium. Furthermore, the synergistic effect was not affected by PKA inhibition. Therefore, our data indicate that CCh induced Ca^2+^-dependent but cAMP/PKA-independent ion secretionin duodenal epithelium, confirming the critical role of pure Ca^2+^ signaling in this process.

We further elucidated the underlying molecular mechanisms by how Ca^2+^ signaling regulates duodenal ion secretion. Although CaCC is critical for Ca^2+^-mediated ion secretion in other epithelial tissues, we did not detect their functional expression in duodenal epithelium. Therefore, the present study focused on the CFTR-mediated ion secretion since we underscored the importance of these channels in the process. Growing evidence suggests that Ca^2+^ signaling can stimulate CFTR channels by activating Ca^2+^-dependent adenylyl cyclase via cAMP/PKA pathway. However, our study ruled out this possibility in the duodenum because cAMP/PKA pathway is not involved in this CCh-induced Ca^2+^-mediatedion secretion. Furthermore, we revealed that Ca^2+^ signaling regulates duodenal ion secretion via a novel PI3K/Akt-mediated activity of CFTR channels.

Compelling evidence shows that cAMP-induced epithelial ion secretion is regulated mainly through PKA-mediated CFTR phosphorylation, which in turn stimulates both channel gating and trafficking to apical plasma membrane of polarized cells, leading to the increase of ion secretion [39, 40]. Previous studies reported the requirement of PI3K for glucagon-induced trafficking of aquaporin-8 [41] and angiotensin II-induced trafficking of Na^+^/H^+^ exchanger to plasma membrane, indicating that PI3K is involved in exocytotic insertion of proteins into plasma membrane [42]. Therefore, we further examined whether intracellular trafficking of CFTR is stimulated by CCh-induced Ca^2+^ signaling. Indeed, CCh stimulated CFTR trafficking to the plasma membranes of villus cells in duodenal epithelia, which was significantly inhibited by PI3K inhibitors. Together, our results obtained from functional, biochemical, and morphological studies have demonstrated the importance of PI3K in the regulation of CCh-mediated duodenal epithelial CFTR channel activity. To provide direct evidence for the role of PI3K in the regulation of duodenal epithelial CFTR function, we further examined PI3K activity and phosphorylation of Akt, a downstream effector of PI3K, in murine duodenal epithelium. Our results indicate that CCh-induced Ca^2+^ signal activates PI3K/Akt in duodenal epithelium.

In conclusion, we underscored the critical role of Ca^2+^ signaling in small intestinal epithelial ion secretion via the SOCE mechanism on the serosal side of epithelial cells. We identified that CRAC/Orai channels may serve as SOCE mechanism to mediate Ca^2+^-dependent epithelial ion secretion. We also demonstrated the molecular mechanisms of Ca^2+^ signaling in CFTR-mediated ion secretion via a novel PI3K/Akt pathway rather than the well-known cAMP/PKA pathway. A scheme summarizes our findings in Figure 6D. A full understanding of Ca^2+^-mediated intestinal epithelial Cl^-^ and HCO_3_^-^ secretion and the precise modulatory mechanisms of this process will greatly enhance our knowledge about ion transports in GI tract. Our findings suggest new perspectives for potential drug targets to protect the upper GI tract through promoting epithelial HCO_3_^-^ secretion and to control intestinal liquid homeostasis through modulating epithelial Cl^-^ secretion.

## MATERIALS AND METHODS

### Cell culture

The HT-29 cell lines used in this work were obtained from the American Type Culture Collection (Rockville, MD) and were cultured at 37°C under 5% CO_2_ in RPMI 1640 medium supplemented with antibiotics (100 U/ml of penicillin and 100μg/ml of streptomycin) and 10% heat-inactivated fetal bovine serum. After the cells had grown to confluence, they were replated onto 12-mm round coverslips (Warner Instruments Inc., Hamden, CT) and incubated for at least 24 h before use for cytosolic Ca^2+^ concentration([Ca^2+^]_cyt_).

### Animal preparation

All studies were approved by Committees on Investigations Involving Animals in Xinqiao Hospital of Third Military Medical University, China and the University of California, San Diego. Experiments were performed with on 6–12 wk male Harlan C-57 black mice; homozygous CFTR knockout (CFTR^-/-^) mice and their wild-type littermates (CFTR^+/+^), which were established as described previously [43].

### Ussing chamber experiments

Ussing chamber experiments were performed as previously described [44]. The duodenal tissue from each animal was stripped of seromuscular layers, divided, and examined in four chambers (window area, 0.1 cm^2^). Experiments were performed under continuous short-circuited conditions (Voltage-Current Clamp, VCC MC6; Physiologic Instruments, San Diego, CA), and luminal pH was maintained at 7.40 by the continuous infusion of 0.5 mM HCl under the automatic control of a pH-stat system (PHM290, pH-Stat controller; Radiometer Copenhagen). The volume of the titrant infused per unit time was used to quantitate HCO_3_^-^ secretion. These measurements were recorded at 5-min intervals. The rate of luminal HCO_3_^-^ secretion is expressed as micromoles per centimeter squared per hour. Transepithelial short-circuit current (*I*_*sc*_; reported as μA or eq cm^-2^ h^-1^) was measured via an automatic voltage clamp. After a 15-30 min measurement of basal parameters, inhibitors were added to the tissues for 10-20 min, as dictated by the experimental design, followed by addition of carbachol (100 μM) or forskolin (10 μM) to the serosal side of tissue.

### Measurement of [Ca^2+^]_cyt_ by digital Ca^2+^ imaging

Ca^2+^ imaging experiments were performed as previously described [45]. HT-29 cells cultured on coverslips were loaded with 5μM Fura-2 AM (Invitrogen, NY, USA) in physiological salt solution (PSS) at 37°C for 50 min and then washed with PSS or PPS with 2-APB (Tocris Bioscience, Minneapolis, MN, USA), a CRAC channel blockers (50μM); or GSK-7975A (Tocris Bioscience, Minneapolis, MN, USA), for 30 min. Then, cells on coverslips were placed in a standard perfusion chamber on the stage of an inverted fluorescence microscope (Nikon, Japan). For the Ca^2+^-free solution, Ca^2+^ was omitted and 0.5 mM EGTA was added to prevent possible Ca^2+^ contamination.

### Phosphate labeling and immunoprecipitation of CFTR

The mice were prepared as described above. Segments of proximal duodenum (∼5 mm) opened along the mesenteric border were placed in modified buffered Ringer solution at 37°C gassed with 95% O_2_-5% CO_2_ for incubation. After stabilization for 20 min, CCh (100 μM) or control vehicle was added to the bathing solution for 5 min of incubation. When wortmannin (0.1 μM) or LY294002 (20 μM) was used, it was added at 30 min before the agents above. Phosphate labeling and immunoprecipitation of CFTR was performed as described [43]. Briefly, CFTR was immunoprecipitated from the supernatant with CFTR polyclonal antibody (CFTR H-182; Santa Cruz Biotechnology) for 1h at 4°C. The samples were centrifuged, and the supernatants were analyzed by 6% SDS-polyacrylamide gel electrophoresis (SDS- PAGE). 32P-phosphorylated CFTR was visualized by autoradiography and quantified by scintillation counting of excised bands.

### Immunoprecipitation of PI3K and ELISA for detection of PI3K and cAMP activity

Segments of murine duodenum were incubated for different time points as described above. Immunoprecipitation of PI3K was performed as described [44]. PI3K activity was measured *in vitro* using a competitive ELISA format (Echelon Biosciences, Salt Lake City, UT) and cAMP activity was measured *in vitro* using Mouse/Rat cAMP assay(FGE012B) from R&D Systems Inc. (Minneapolis, MN, USA) according to the manufacturer’s instructions. Enzyme activity was estimated by comparing the values from samples containing enzymatic reaction products to the values in the standard curve.

### Western blot analysis for measurement of Akt phosphorylation

Segments of murine duodenum were incubated with CCh (100 μM), or control vehicle for different time points as described above. At the end of incubation, the tissue was processed as described in detection of PI3K activity above. Protein concentrations were determined using a Bradford protein assay (BioRad, Hercules, CA, USA). A total of 50 μg protein aliquots were separated by SDS-PAGE electrophoresis and blotted using a V3 Western Workflow system (BioRad, Hercules, CA, USA) according to the manufacturer’s instructions. PVDF membranes were incubated with primary antibodies: anti-phospho-Akt S473 or anti-Akt (diluted 1:1,000; Cell Signaling) overnight at 4°C. All results were measured by densitometry and presented as relative expression to tubulin as a reference protein.

### Immunohistochemistry

Segments of murine duodenum were incubated as described above. Immunofluorescence labeling was performed as described [43]. Sections were incubated in a moist chamber overnight with primary anti-CFTR antibody (CFTR H-182, 1:50 dilution in 1% PBS-BSA) at 4°C, washed three times for 15 min each and incubated with FITC-conjugated secondary antibody (affinity purified donkey anti-rabbit IgG, 1:400 dillution in 1% PBS-BSA), for 1 h at room temperature to detect CFTR staining. Confocal microscopy and images analysis of CFTR fluorescence intensities were performed as described by Ameen et al. [46, 47] using a confocal microscope (TCS SP2 AOBS; Leica, Wetzlar, Germany) equipped with image analysis software. Data were collected from an average of 30 cells in random sections labeled for CFTR (average 10 sections) from each tissue examined (4 animals in each series) and is expressed as the ratio of plasma membrane to cytoplasm fluorescence intensity.

### Statistics

The data and statistical analysis comply with the recommendations on experimental design and analysis in pharmacology [48]. All results are means ± SE. Net peaks for duodenal HCO_3_^-^ secretion and *I*_*sc*_ refer to stimulated peak responses minus basal levels. Data were analyzed using one-way analysis of variance followed by the Newman-Keul post hoc test or, when appropriate, by the two-tailed Student’s *t*-tests. P<0.05 was considered statistically significant.

### Materials

The following drugs were used: carbachol, Forskolin, wortmannin, LY294002, and rapamycin from Sigma; CFTRinh-172 from Calbiochem; [^32^P]orthophosphate from Amer-sham; Anti-PI3K p85 was obtained from Upstate Biotechnology; Anti-phospho-Akt S473 antibody and anti-Akt antibody were obtained from Cell Signaling; All other chemicals in solutions were obtained from Sigma and Calbiochem.

## SUPPLEMENTARY MATERIALS FIGURES



## Abbreviations

CCh: carbachol
CFTR: cystic fibrosis transmembrane conductance regulator
GI: gastrointestinal
CaCCs: Ca^2+^-activated Cl^-^ channels
2-APB: 2-aminoethoxydiphenyl borate
SOCE: store-operated Ca^2+^ entry
CRAC: Ca^2+^ release-activated Ca^2+^ channels
IP_3_: inositol 1,4,5-trisphoshate
[Ca^2+^]_cyt_: cytosolic Ca^2+^ concentration
IEC: intestinal epithelial cells
*I*_sc_: short-circuit current
FFA: Flufenamic acid
ER: endoplasmic reticulum
NFA: niflumic acid
VGCC: voltage-gated Ca^2+^ channels
IRBIT: IP3R binding protein released with inositol 1,4,5-trisphosphate
