# Supplementary material for: Molecular mechanisms of calcium signaling in the modulation of small intestinal ion transports and bicarbonate secretion
Source: Oncotarget. 2017 Dec 11;9(3):3727–40. doi: 10.18632/oncotarget.23197 (PMC5790495; doi:10.18632/oncotarget.23197)
Supplement: Supplementary file 1 [file oncotarget-09-3727-s001.pdf]

Molecular mechanisms of calcium signaling in the modulation of small intestinal ion transports and bicarbonate secretion

SUPPLEMENTARY MATERIALS

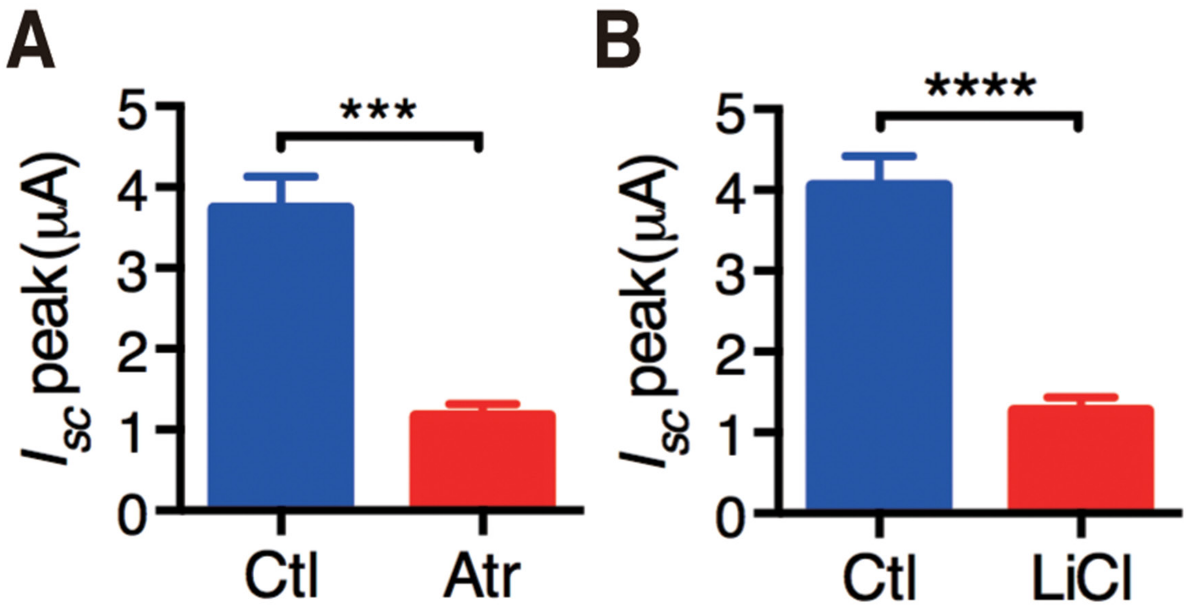

Supplementary Figure 1: Activation of muscarinic receptors induced intracellular Ca^{2+} release. (A-B) Effects of atropine (Atr, 10μM) (**A**) and LiCl (30mM) (**B**) on CCh-induced duodenal I_{sc} peak after both serosal and mucosal addition. Results are presented as mean±SE (n = 6–9 in each series). $^{****}P < 0.0001$ vs. control or DMSO by Student's t-test.

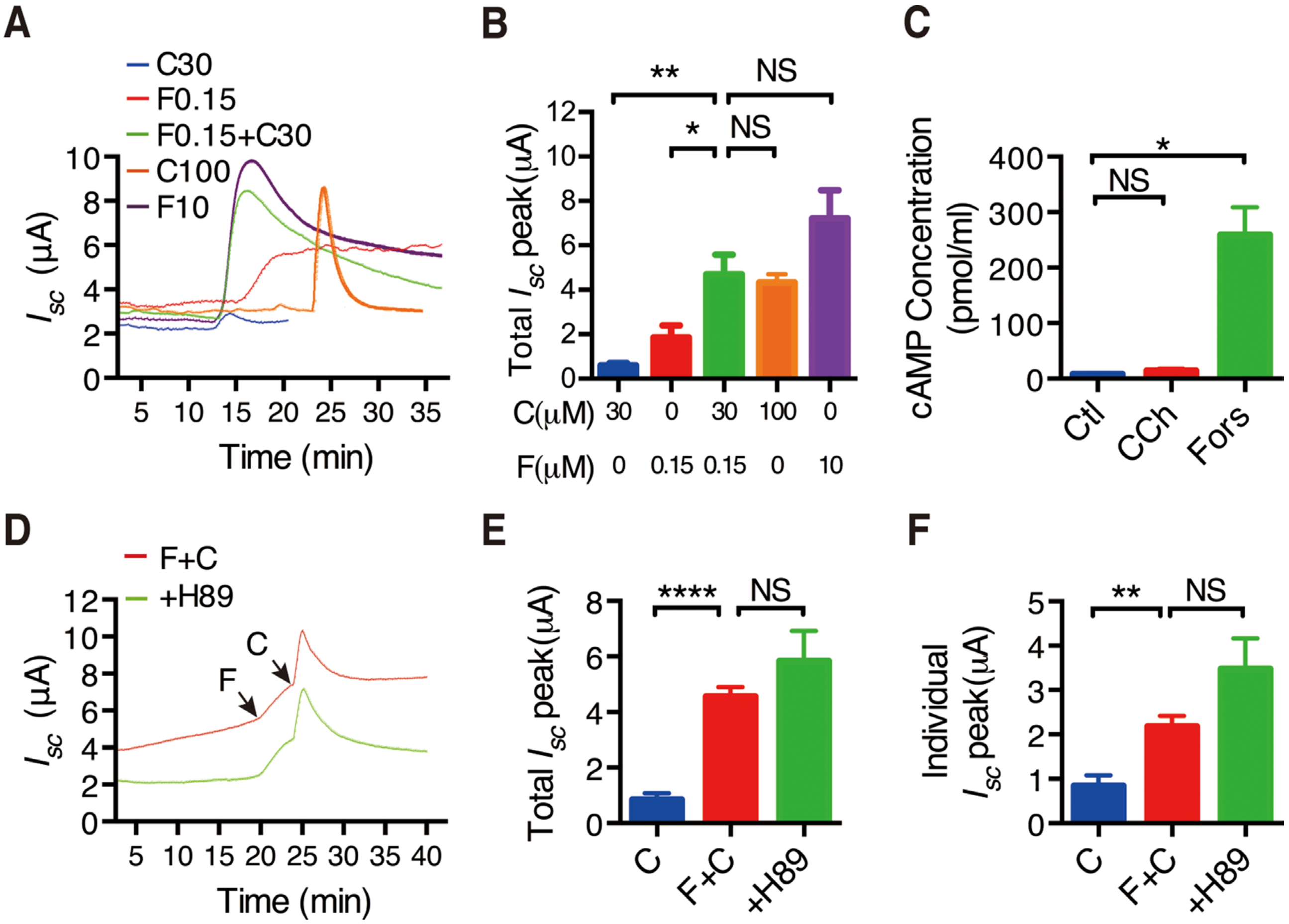

Supplementary Figure 2: CCh-induced duodenal ion secretion was cAMP/PKA-independent. (**A**–**B**) Time course of stimulating murine duodenal epithelial with carbachol (C, 30μM or 100μM), Forskolin (F, 0.15μM or 10μM) or both CCh (30μM) and Forskolin (0.15μM). The summary data of CCh or Forskolin-stimulated duodenal I_{sc} peak of (A) is shown as (B) ($n = 5$). (**C**) CCh (100μM) or Forskolin (10μM)-stimulated duodenal mucosal epithelial cAMP activity ($n = 3$). (**D**) Representative of the time course of adding Forskolin (0.15μM) followed by CCh (30μM) and the effect of the PKA inhibitor H89 (20μM) added to both serosal and mucosal sides before the Forskolin. (**E**) Summary on the effect of H89 (20μM) on CCh (30μM) together with Forskolin (0.15μM)-stimulated duodenal I_{sc} peak of (D) ($n = 6$). (**F**) Summary on the effect of H89 (20μM) on individual CCh (30μM)-stimulated duodenal I_{sc} peak of (D) ($n = 6$). Results are presented as mean ± SE. NS, no significant differences, $^{*}P < 0.05$, $^{**}P < 0.01$ or $^{****}P < 0.0001$ *vs*. control or DMSO.
